# Role of Jumonji domain-containing protein D3 and its inhibitor GSK-J4 in Hashimoto’s thyroiditis

**DOI:** 10.1515/med-2023-0659

**Published:** 2023-03-01

**Authors:** Xixuan Lu, Ying Liu, Li Xu, Haiyan Liang, Xiaoli Zhou, Hong Lei, Liping Sha

**Affiliations:** Department of Endocrinology, Cardiovascular and Cerebrovascular Disease Hospital, General Hospital of Ningxia Medical University, No. 804, Shengli South Street, Xingqing District, Yinchuan 750004, Ningxia, China; Department of Radiology, The 942th Hospital of the People’s Liberation Army Joint Logistics Support Force, Yinchuan, Ningxia, China; Department of Endocrinology, Cardiovascular and Cerebrovascular Disease Hospital, General Hospital of Ningxia Medical University, Yinchuan 750004, Ningxia, China

**Keywords:** Hashimoto thyroiditis, JMJD3, GSK-J4, epigenetics, autoimmunity

## Abstract

Hashimoto’s thyroiditis (HT) is an autoimmune illness caused by a combination of genetic, epigenetic, and environmental factors. The pathogenesis of HT is not fully elucidated, especially in epigenetics. The epigenetic regulator Jumonji domain-containing protein D3 (JMJD3) has been extensively investigated in immunological disorders. This study has been performed to explore the roles and potential mechanisms of JMJD3 in HT. Thyroid samples from patients and healthy subjects were collected. We first analyzed the expression of JMJD3 and chemokines in the thyroid gland using real-time PCR and immunohistochemistry. *In vitro*, the apoptosis effect of the JMJD3-specific inhibitor GSK-J4 on the thyroid epithelial cell line Nthy-ori 3-1 was evaluated using FITC Annexin V Detection kit. Reverse transcription-polymerase chain reaction and Western blotting were applied to examine the inhibitory effect of GSK-J4 on the inflammation of thyrocytes. In the thyroid tissue of HT patients, JMJD3 messenger RNA and protein levels were substantially greater than in controls (*P* < 0.05). Chemokines C–X–C motif chemokine ligand 10 (CXCL10) and C–C motif chemokine ligand 2 (CCL2) were elevated in HT patients, and thyroid cells with stimulation of tumor necrosis factor α (TNF-α). GSK-J4 could suppress TNF-α-induced synthesis of chemokines CXCL10 and CCL2 and prohibit thyrocyte apoptosis. Our results shed light on the potential role of JMJD3 in HT and indicate that JMJD3 may become a novel therapeutic target in HT treatment and prevention.

## 1 Introduction

Hashimoto’s thyroiditis (HT) is a common endocrine disease marked by the diffuse infiltration of immune cells in the thyroid gland, which generate a mass of autoantibodies directed against thyroid-specific antigens, including thyroglobulin (TG) and thyroperoxidase (TPO) [1]. Although the precise pathogenesis of HT is not fully understood, it is presently thought that, in some genetic backgrounds, a combination of environmental stimuli (e.g., iodine and viruses) and epigenetic alterations leads to autoimmune thyroid intolerance [2].

The nuclear factor kappa-beta (NF-κB) signaling pathway also plays a vital role in HT. Activated inflammatory cells such as macrophages and lymphocytes can produce a large number of cytokines, such as tumor necrosis factor α (TNF-α) and interferon γ (IFN-γ) [3]. TNF-α and IFN-γ, in addition to promoting inflammatory cells interaction and affecting the differentiation and activation of lymphocytes, can induce thyrocytes to express a variety of chemokines and adhesion molecules, such as C–X–C motif chemokine ligand 10 (CXCL10) and C–C motif chemokine ligand 2 (CCL2), resulting in a cascade of inflammation that aggravates thyroid damage. The immune response requires the action of adhesion molecules and chemokines [4,5]. Jumonji domain-containing protein D3 (JMJD3) is a member of the UTX/UTY JmjC-domain protein subfamily that specifically demethylates di- and trimethyl-lysine 27 on histone H3 (H3K27me2/3) [6]. JMJD3 has been widely studied in immune diseases, infectious diseases, cancer, developmental diseases, and aging-related diseases [7]. JMJD3 deficient mice are reported to be resistant to experimental autoimmune encephalomyelitis [8]. A recent study pointed out that there was a high expression of JMJD3 on CD4 + T cells accompanied by a significant decrease in H3K27me3 in patients with systemic sclerosis [9]. In systemic lupus erythematosus, decreased JMJD3 binding in the promoter with increased H3K27me3 marks is associated with the low expression of hematopoietic progenitor kinase 1, which negatively regulates the activation of T cells [10]. However, the role of JMJD3 in HT remains unclear. This present study aimed to explore the roles and potential mechanisms of JMJD3 in HT.

## 2 Methods

### 2.1 Subjects

Thyroid specimens were obtained from the non-affected lobes of patients who underwent thyroidectomy because of thyroid cancer or thyroid adenoma with a cytological diagnosis of cancer. Thyroid antibodies were detected, and thyroid function was measured using an electrochemiluminescence technique utilizing serum samples. Patients in the HT group were positive for at least one thyroid antibody (TG-Ab and TPO-Ab) and had pathologically confirmed HT during surgery. Subjects in the control group were age-matched and were negative for both thyroid antibody and pathological diagnosis of HT. All the participants were euthyroid at the time of surgery and did not have any systemic autoimmune disease, severe disease, thyroid hormone replacement, or anti-thyroid medication. The clinical characteristics of patients and controls are listed in Tables A1 and A2.

### 2.2 Cell culture and stimuli

The normal human thyroid epithelial cell line Nthy-ori 3-1 was given generously by the First Affiliated Hospital of China Medical University, China. Cells were cultured in RPMI 1640 medium (Invitrogen, Carlsbad, CA, USA) containing 10% fetal bovine serum (Gibco, Grand Island, NY, USA) and 1% penicillin–streptomycin, incubated at 37°C with 5% carbon dioxide. The inflammation model of Nthy-ori 3-1 was constructed with the stimulation of TNF-α (20 ng) (Sigma, St. Louis, MO, USA) and varied doses of sodium iodide (NaI; Sigma) and co-stimulated with JSK-J4 (20 µmol; Selleck, Houston, TX, USA) for 6 and 24 h.

### 2.3 Western blotting

Cultured cells were lysed on ice with 100 µl lysis buffer, collected by a cell scraper for a violent vortex, and then centrifuged at 4°C at 12,000 rpm for 10 min. Supernatants were collected, and the quantified proteins were boiled for sodium dodecyl sulfate polyacrylamide gel electrophoresis. Samples were run on 6 and 12% Tris-Glycine gels separately. Immunoblotting was performed according to standard protocol, transferred to polyvinylidene fluoride membrane (Merck Millipore, Billerica, MA, USA), blocked with tris buffered saline with tween (TBST) + 5% skim milk for 2 h at room temperature, incubated overnight at 4 °C with the following antibodies: anti-CCL2 (Abcam, #9669, Cambridge, MA, USA), anti-β-actin (Abcam, #8226), and anti-CXCL10 (Abcam, #9807). After incubating with the aforementioned antibodies, samples were washed three times with horseradish peroxidase and incubated with HRP-conjugated IgG antibodies for 2 h at room temperature. Specific bands were detected using the chemiluminescent substrate (Thermo Fisher, Waltham, MA, USA). Immunoblot bands were quantified in relation to β-actin using Image-Pro Plus.

### 2.4 Real-time PCR

For gene expression analysis in thyroid tissues, another new cohort of 60 subjects (30 controls and 30 HT) meeting the same criteria mentioned earlier was included to detect JMJD3. Total RNA from cell lines and tissue samples was extracted using Trizol reagent (Invitrogen). Reverse transcription was achieved using Takara Kit RR047A (TaKaRa, Dalian, China). Complementary DNA amplification was then performed using SYBR Premix Ex Taq (TaKaRa, RR820) in the Roche 480 LightCycler. The mRNA expression for each sample was analyzed using glyceraldehyde-3-phosphate dehydrogenase (GAPDH) as an internal control. The sequences of the primers used for amplification were as follows: CCL2 forward, 5′-ACTCTCGCCTCCAGCATGAA-3′ and reverse, 5′-TTGATTGCATCTGGCTGAGC-3′ (TaKaRa); CXCL10 forward, 5′-GCTTCCAAGGATGGACCACA-3′ and reverse, 3′-GCAGGGTCAGAACATCCACT-5′ (TaKaRa); JMJD3 forward, 5′-GCAGGAATGCCAAGGTGAAAG-3′ and reverse, 5′-GCAGCAGGACAGGTGAGAAGG-3′; and GAPDH forward, 5′-TGACAACTTTGGTATCGTGGAAGG-3′ and reverse, 5′-AGGCAGGGATGATGTTCTGGAGAG-3′. Data processing used the 2^−ΔΔCT^ method.

### 2.5 Immunohistochemistry

Paraffin-embedded thyroid tissue sections were used to detect the JMJD3 protein. After dewaxing and hydration, paraffin sections were incubated with 3% H_2_O_2_ for 15 min, placed in a microwave oven for a total of 10 min for antigen retrieval, blocked with goat serum for 20 min, and then incubated with JMJD3 antibody (Abcam, #38113) overnight. After incubation with a biotin-labeled secondary antibody, diaminobenzidine was added for color. Then, the sections were stained with hematoxylin for 5 min, placed in gradient alcohol for dehydration, and observed using a microscope (Olympus, Tokyo, Japan) after sealing.

### 2.6 Apoptosis assay

A total of 2  ×  10^5^ cells/well in six-well plates were exposed to TNF-α and JSK-J4 and then harvested at the indicated time points. According to the manufacturer’s instructions, the apoptosis assay was conducted using the FITC Annexin V Apoptosis Detection Kit (BD Biosciences, San Jose, CA, USA).

### 2.7 Statistical analysis

SPSS Software 20.0 was used for statistical analysis (SPSS, Inc., Chicago, IL, USA). For normally distributed data, the results were presented as means with standard deviations, and the difference between the patient and control groups was compared using an independent sample *T*-test. Non-normally distributed data are expressed as the median (inter-quartile range), and the rank sum test was used to detect differences. The data were analyzed, and graphics were created using the GraphPad Prism 6 program. A *P*-value of <0.05 was considered statistically significant.


**Ethics approval:** This study was approved by the Ethics Committee of the Ningxia Medical University General Hospital, and all procedures involving human volunteers were carried out in compliance with the ethical standards of the 1964 Declaration of Helsinki and its later amendments.

## 3 Results

### 3.1 JMJD3 was elevated in the thyroid tissue of patients with HT

**Figure 1:**
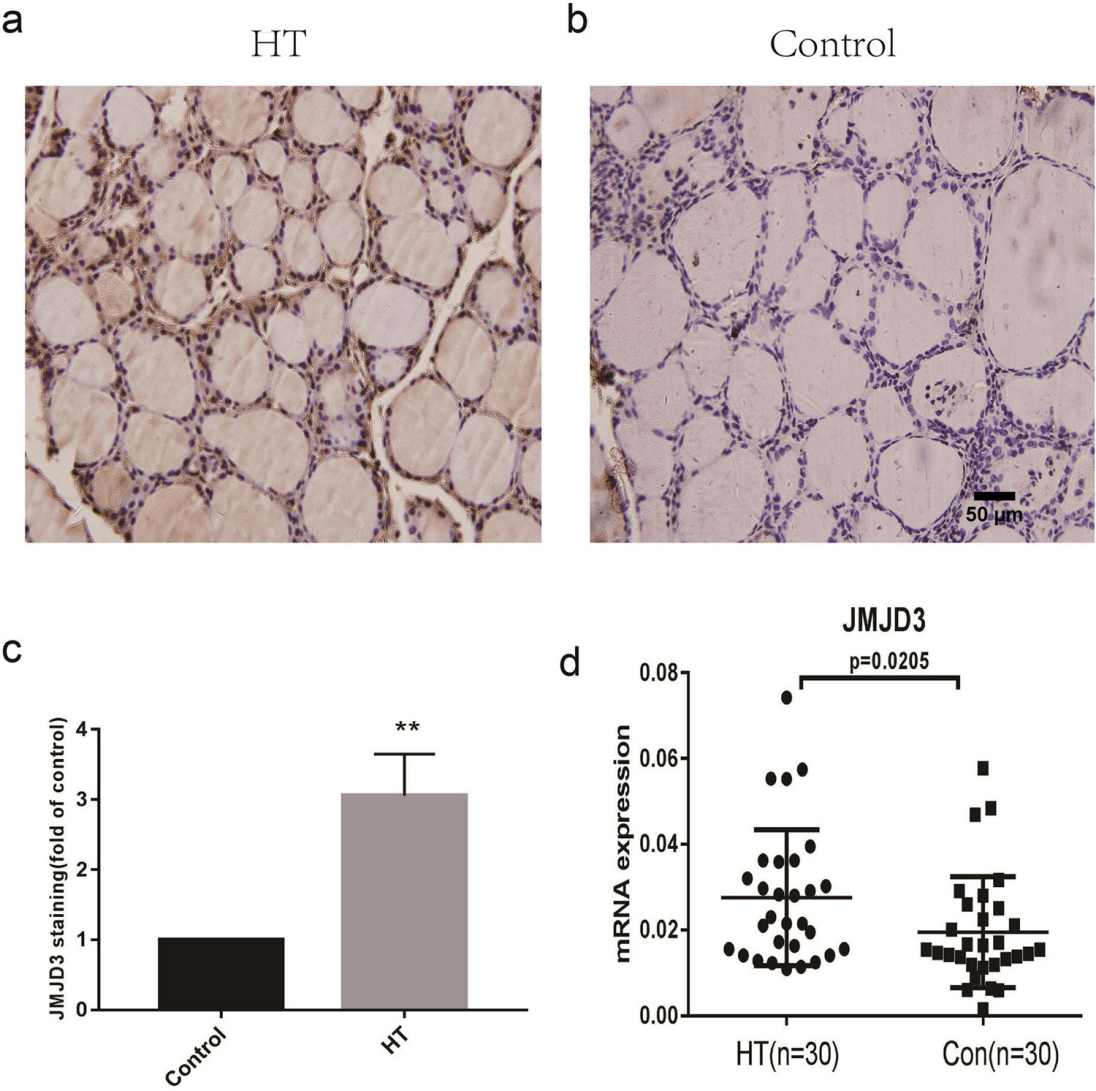
Overexpression of JMJD3 in the thyroid tissue of HT patients. Con: control. (a) Immunohistochemical to detect JMJD3 in thyroid gland from HT patients (*n* = 6). (b) Immunohistochemical to detect JMJD3 in age-matched control patients (*n* = 6). (c) Histogram to show protein level of JMJD3. (d) JMJD3 mRNA expression in HT and control groups (*n* = 60). **P < 0.01 vs control.

We used PCR (30 HT vs 30 controls) and immunohistochemistry (6 HT vs 6 controls) to determine the expression level of JMJD3 in HT thyroid tissue and discovered that the mRNA and protein levels of JMJD3 in HT thyroid tissue were substantially higher than in controls (*P* < 0.01), as shown in Figure 1.

### 3.2 Increased expression of CCL2 and CXCL10 in the thyroid tissue of HT patients

**Figure 2:**
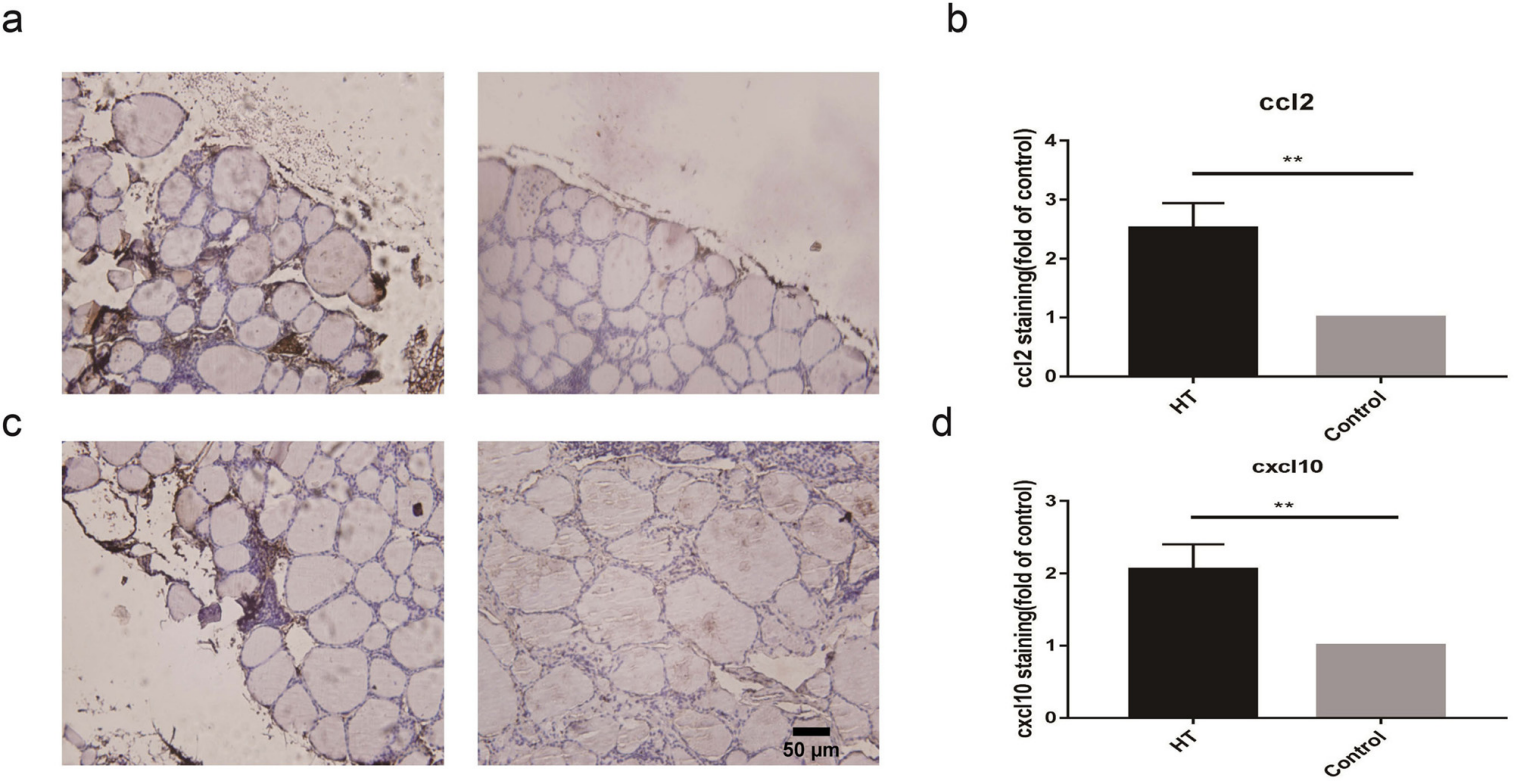
Enhanced expression of CCL2 and CXCL10 in HT patients. (a) Immunohistochemistry to detect CCL2 in thyroid gland from HT patients (*n* = 6) and the control group (*n* = 6). (b) Histogram of CCL2 protein level. (c) Immunohistochemistry to detect CXCL10 in HT patients (*n* = 6) and the control group (*n* = 6). (d) Histogram of CXCL10 protein level. **P < 0.01 vs control.

We used immunohistochemistry (6 HT vs 6 controls) to confirm the protein expression of CCL2 and CXCL10 in the thyroid and found that HT patients had significantly higher levels of CCL2 and CXCL10 than controls (*P* < 0.01), as illustrated in Figure 2.

### 3.3 GSK-J4 inhibited the expression of CCL2 and CXCL10 in the presence of TNF-α

**Figure 3:**
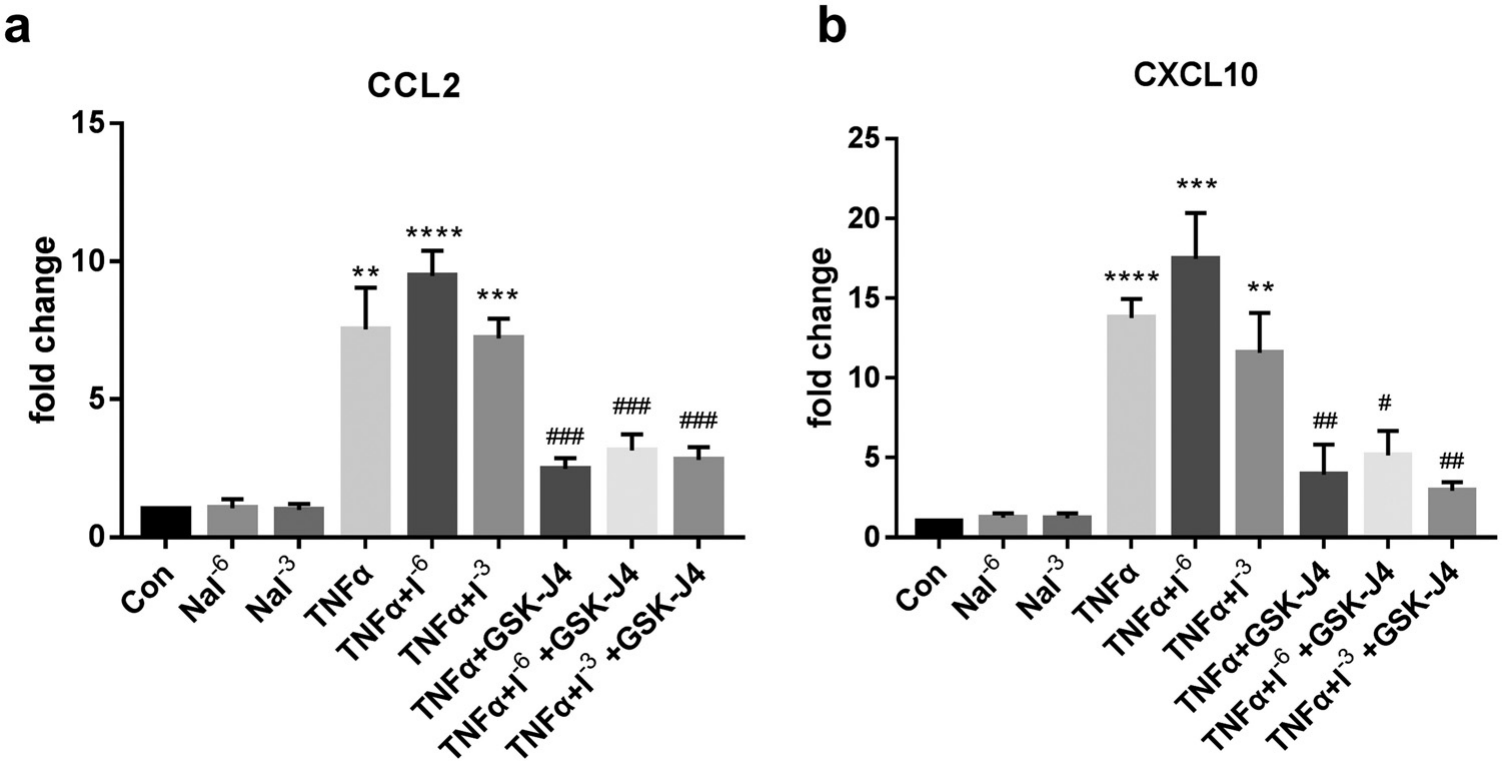
The expression of CCL2 and CXCL10 in thyroid cells with stimuli. Con: control. NaI^−6^/I^−6^: 10^−6^ mol/L NaI; NaI^−3^/I^−3^: 1 × 10^−3^ mol/L NaI. (a) mRNA expression of CCL2 with the stimulation of TNF-α, iodine, and GSK-J4. (b) mRNA expression of CXCL10 with the stimulation of TNF-α, iodine, and GSK-J4. **P < 0.01, ***P < 0.001, ****P < 0.0001 vs control group; ^#^ *P* < 0.05, ^##^ *P* < 0.01, ^###^ *P* < 0.001 vs TNF-α stimulation without GSK-J4.

**Figure 4:**
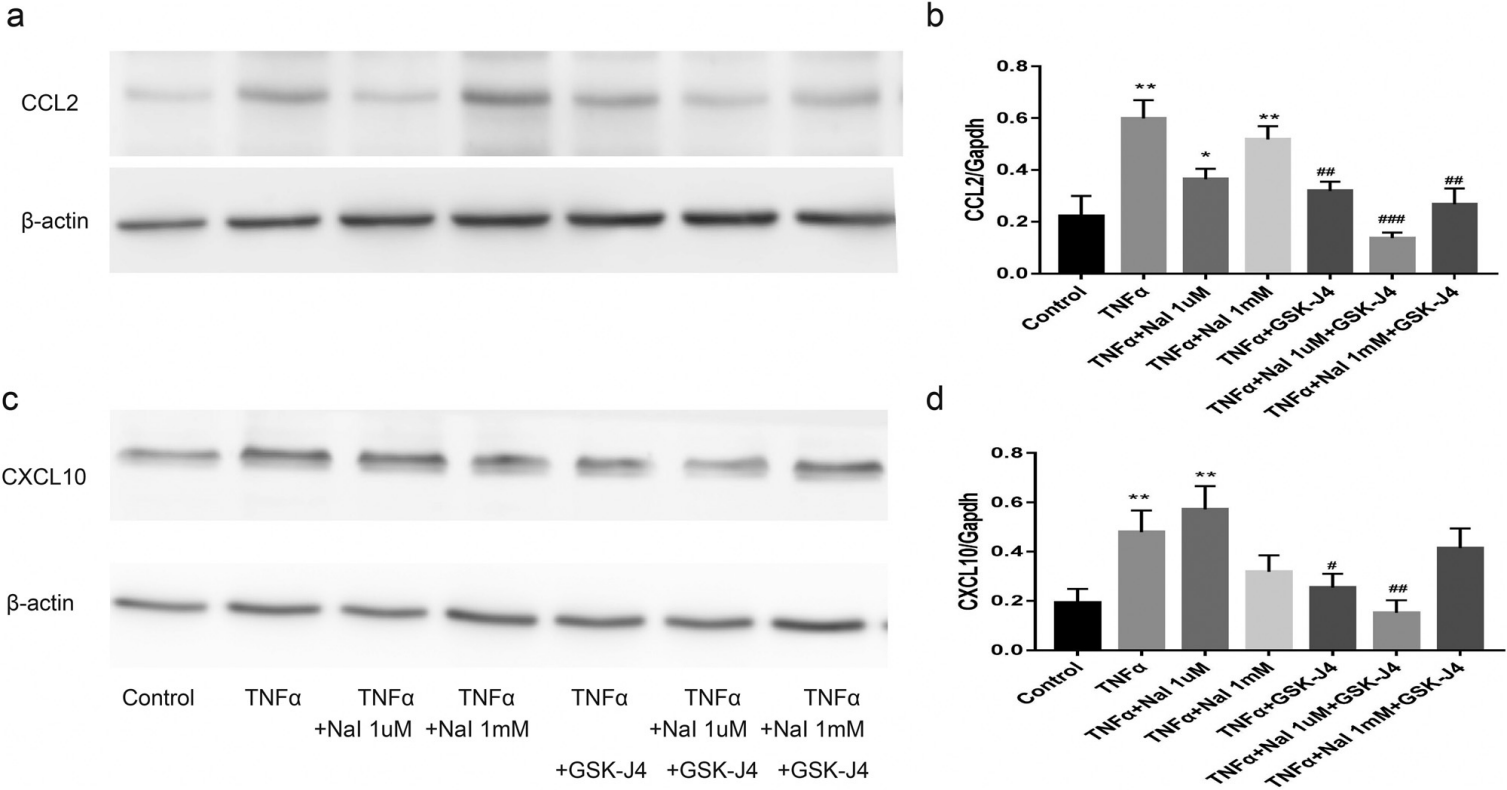
The effects of GSK-J4 on the chemokines of thyroid cells under the action of TNF-α. (a) WB to evaluate Protein expression of CCL2. (b) Histogram representation of CCL2 protein levels. (c) WB to evaluate Protein expression of CXCL10. (d) Histogram representation of CXCL10 protein levels. *P < 0.05, **P < 0.01 vs control group; ^#^ *P* < 0.05, ^##^ *P* < 0.01, ^###^ *P* < 0.001 vs TNF-α stimulation without GSK-J4.

Previous studies have shown that iodine has been linked to thyrocyte inflammation. The transcriptional expression of CCL2 and CXCL10 in Nthy-ori 3-1 did not alter the following stimulation with different doses of iodine in this investigation (*P* > 0.05). TNF-α (20 ng) activation, on the other hand, resulted in a substantial upregulation of CCL2 and CXCL10 mRNA levels (*P* < 0.05). Iodine had no synergistic effect with TNF-α on thyroid cells (*P* > 0.05). When thyroid cells were co-stimulated with JMJD3 inhibitor GSK-J4 and TNF-α, the mRNA levels of CCL2 and CXCL10 dropped considerably (*P* < 0.01), as shown in Figure 3. Western blotting (WB) was applied to verify the protein levels of CCL2 and CXCL10. As we can see from Figure 4, CCL2 and CXCL10 were clearly downregulated (*P* < 0.05) after the treatment of GSK-J4, which is consistent with the results of mRNA.

### 3.4 GSK-J4 reduced thyrocyte apoptosis in the presence of TNF-α

**Figure 5:**
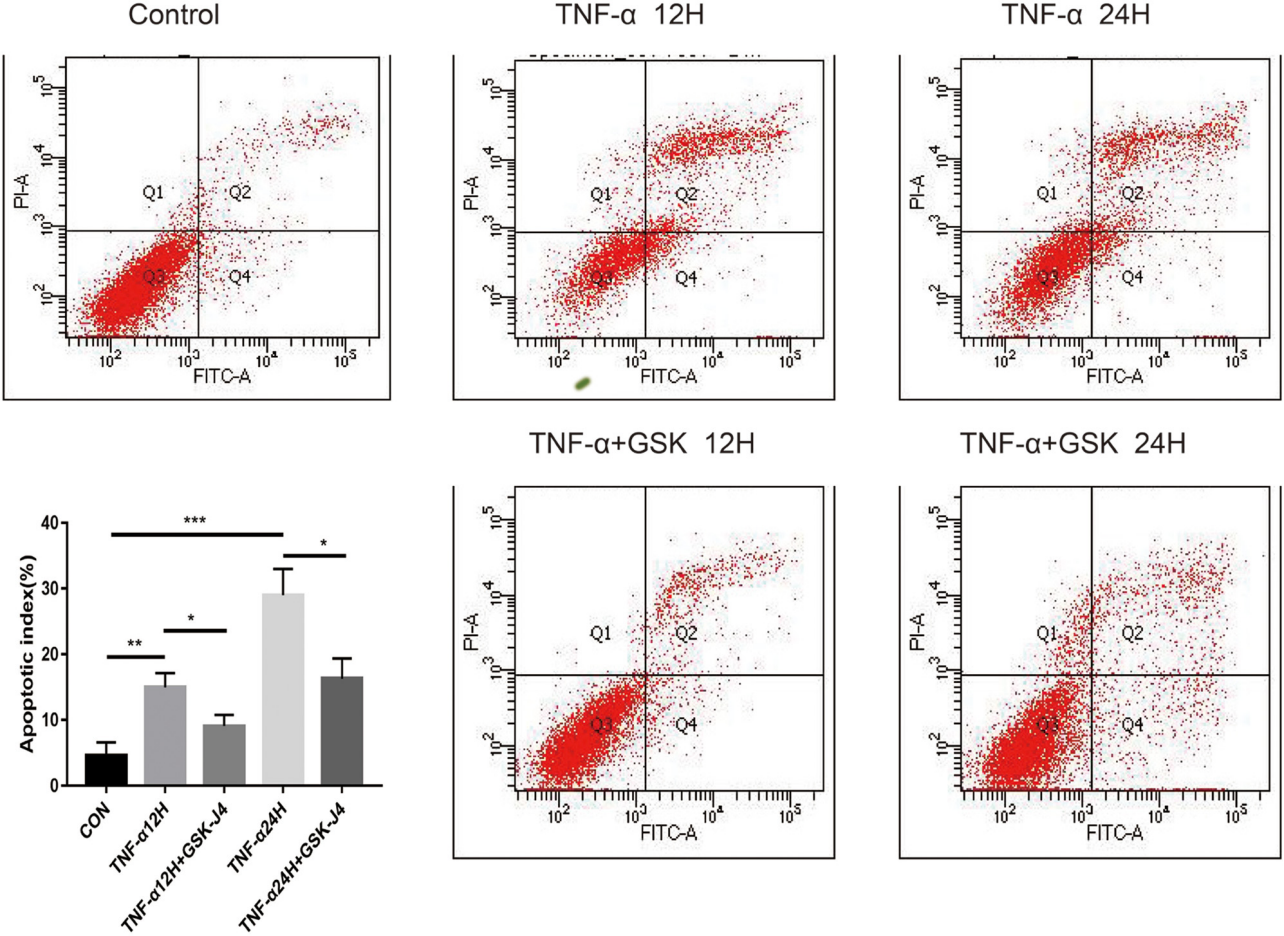
The impact of GSK-J4 on thyroid cells apoptosis with the stimulation of TNF-α. *P < 0.05, **P < 0.01, ***P < 0.001 vs control.

Thyroid cell apoptosis rose significantly in response to TNF-α stimulation, and the apoptosis level increased progressively as the stimulation time increased. When GSK-J4 was combined, thyrocyte apoptosis was dramatically decreased at each stimulation period, as may be seen in Figure 5.

## 4 Discussion

In the present study, we observed that JMJD3 was considerably elevated in the thyroid of HT patients. The JMJD3 inhibitor GSK-J4 downregulated the expression of CCL2 and CXCL10 and inhibited thyroid cell apoptosis *in vitro*.

JMJD3 has been discovered to have an essential epigenetic modification in the development and differentiation of tumors and metabolism. JMJD3 also plays an important regulatory role in the inflammatory and immune systems. Pro-inflammatory cytokines caused JMJD3 to translocate to the transcription start sites of hundreds of activated genes in the nucleus [11]. Meanwhile, by targeting certain transcription factors, JMJD3 can increase pro-inflammatory responses [12]. JMJD3 has been identified as a critical modulator of the NF-κB pathway and a potential therapeutic target for NF-κB-related diseases, including atherosclerosis [13].

JMJD3 can boost the transcriptional expression of inflammatory genes through NF-κB, signal transducers and activators of transcription, SMAD pathways, and T-bet transcription factors [14]. According to recent research, increased JMJD3 expression on CD4^+^ T cells in individuals with systemic sclerosis was associated with a large drop in H3K27me3 [9]. JMJD3 is also involved in the regulation of the differentiation of CD4^+^ T cells. JMJD3 rose dramatically after CD4^+^ T cell activation, whereas knocking down JMJD3 greatly inhibited CD4^+^ T cell activation [8]. JMJD3 acts on the promoter of the Rorc gene to decrease the inhibitory H3K27me3, allowing T cells to differentiate into T-helper 17 cells and enhance the production of interleukin (IL)-17 and IL-22 [8]. When JMJD3 is knocked out in human leukemia monocytes (THP-1) cells, the expression of key inflammation markers, such as heat shock protein β-1 (HspB1), increased triple motif protein (TRIM5), and glutathione Glycine peroxidase (Gpx), is dramatically decreased. The changes in these inflammatory markers are related to NF-κB, chemokines, and CD40 signaling pathways. JMJD3 knockout inhibits NF-κB-regulated inflammatory genes [15]. Serum amyloid A protein stimulates macrophages to overexpress JMJD3 and reduce the level of H3K27me3, which contributes to the development of atherosclerotic diseases. Inhibiting the expression of JMJD3 can prevent macrophages from secreting pro-inflammatory cytokines [16].

The essence of HT is an autoimmune response. JMJD3 was identified to be substantially expressed in HT patients for the first time. We used GSK-J4 to intervene further in order to better understand its immunological mechanism in HT. GSK-J4, a JMJD3-specific pharmacological inhibitor, allows JMJD3 to be targeted to treat a variety of related diseases. GSK-J4 was reported to attenuate lipopolysaccharide-induced pro-inflammatory cytokine production *in vitro* [17]. Another research suggested that GSK-J4 could inhibit IL-1β-induced degradation of collagen II and suppress IL-1β-stimulated NF-κB signal pathway activation *in vitro*. Meanwhile, GSK-J4 prevented cartilage damage in a mouse DMM-induced osteoarthritis model *in vivo* [18]. GSK-J4 has been shown to protect islet cells from inflammation-induced apoptosis by inhibiting the NF-κB signaling pathway while upregulating the expression of genes involved in islet function and boosting insulin secretion [19]. TNF-α increased the production of inflammation-related molecules CCL2 and CXCL10 in thyroid cells *in vitro*, as shown by our results, which are comparable to prior findings [20,21,22]. When GSK-J4 was co-treated, the expression of CCL2 and CXCL10 was attenuated. CCL2 and CXCL10 are classic chemokines that can chemoattract lymphocytes and monocyte macrophages to infiltrate target organs, which are involved in the initiation of immune response and acceleration of the inflammation development and are related to many autoimmune diseases, including HT [23]. Previous studies have shown that the expression of CCL2 and CXCL10 is increased in thyroid tissue [24,25], which is consistent with our results. CCL2 and CXCL10 can be induced by pro-inflammatory factors and iodine to increase their expression significantly *in vitro*, indicating that CCL2 and CXCL10 are engaged in the incidence and progression of autoimmune thyroid disorders.

JMJD3 is considered a critical regulator of cytokine production in human NK cell subsets. GSK-J4 increased global levels of the repressive H3K27me3 mark around the transcription start sites of cytokine genes. Moreover, in cytokine-stimulated NK cells, GSK-J4 lowered IFN-γ, TNF-α, granulocyte-macrophage colony-stimulating factor, and IL-10 levels, suggesting its wide applicability in regulating immunological and inflammatory responses [26]. GSK-J4 may help to prevent inflammatory colitis by lowering the inflammatory potential of DCs and enhancing their tolerogenic properties. Mechanistic analyses revealed that GSK-J4 has an impact on regulatory T cells (Treg), increasing their lineage stability and gut tropism as well as potentiating their suppressive activity through increased epigenetic regulation [27]. Silencing JMJD3 or applying GSK-J4 to inhibit the activity of JMJD3 can drastically downregulate inflammatory factors induced by IL-1β by affecting the level of H3K27me3 in the promoter in joint fibroblasts of patients with rheumatoid arthritis [28]. GSK-J4 also slowed down the symptoms of the collagen-induced mouse arthritis model *in vivo* [28]. In pancreatic islet cells, GSK-J4 can inhibit the expression of CCL2 and other inflammatory factors stimulated by IFN-γ, TNF-α, and IL-1β [19], which is in line with our results. Das et al. revealed that reducing the expression of JMJD3 can sharply reduce the transcription of a variety of inflammatory genes in THP-1 macrophages, including cytokines, chemokines, and immune receptors with elevated levels of inhibitory H3K27me3 in their promoters [15]. This suggests that GSK-J4 may regulate the expression of TNF-α downstream inflammatory factors, including CCL2 and CXCL10, by inhibiting the activity of JMJD3 and increasing the level of H3K27me3 in their promoters, which may decrease the interaction between thyroid cells and inflammatory cells and slow down the occurrence and development of HT. GSK-J4 appears to have significant research potential for autoimmune disorders. Our findings also lay the groundwork for additional research into GSK-J4 in HT animal models.

In addition, we demonstrated that GSK-J4 could prevent thyroid cell apoptosis induced by TNF-α. A similar study suggested that GSK-J4 could prevent the apoptosis of human and mouse pancreatic islet cells *in vitro* by reducing the level of inducible nitric oxide synthase in cells [29]. Another research found that JMJD3 has an anti-apoptotic effect on osteoblasts. JMJD3 knockout causes downregulation of B-cell lymphoma-2 to promote apoptosis [30]. It can be seen that the role of JMJD3 in apoptosis is cell-specific, and the specific mechanism of GSK- J4’s anti-apoptosis needs further research. However, our results manifested that GSK J4 might have a particularly protective effect on thyroid cells under inflammatory stimulation.

Earlier literature suggested that high iodine could induce thyrocytes to express a variety of inflammation-related molecules, such as CCL2 and CXCL10 [31], which participated in the pathological process of HT. However, in this study, we failed to find the immune stimulation of iodine in normal thyroid cell lines. It is suggested that the involvement of high iodine in thyroid autoimmunity may require the interaction of genetic background, and the effects of iodine on the autoimmunity of thyrocytes may be based on the mutual activation of thyrocytes and inflammatory cells, which requires more profound mechanism research.

## 5 Conclusions

Collectively, JMJD3 is elevated in the thyroid of HT patients, according to the findings of this study. The specific JMJD3 inhibitor GSK-J4 suppresses the expression of chemokines CCL2 and CXCL10 in thyroid cells and prevents thyrocyte apoptosis. Our findings provide a foundation for the epigenetic research of HT and point to a new direction for HT prevention and treatment. This experiment has only involved an *in vitro* study; more in-depth research will be needed to confirm and further explore the molecular and functional mechanisms.

## Appendix

**Table A1:** The clinical characteristics of patients with Hashimoto’s thyroiditis and controls used in RT-PCR

Variable	HT	Control	Reference Range	*P*-value	
Number.	30	30	—	—	
Age^a^	49.20±2.05	52.17±2.84	—	0.21	
Gender(M/F)	6/24	6/24	—	—	
TSH(mIU/L)^b^	2.47(1.68,3.26)	2.10(1.46,3.74)	0.35–4.94	0.378	
FT4(pmol/L)	12.56±1.42	13.98±1.86	9.01–19.05	0.114	
FT3(pmol/L)	3.57±0.43	4.23±1.01	2.63–5.70	0.268	
TPOAb(IU/mL)	135.8(65.15,558.7)	0.48(0.30,0.76)	0–5.61	0.000***	
TgAb(IU/mL)	100.7(19.75,514.9)	1.43(0.92,2.04)	0–4.11	0.000***	

HT: Hashimoto’s thyroiditis. a. Age, FT4, and FT3 were normally distributed and were expressed as Mean±SDs. Mean comparison between the two groups was performed with the independent sample T-test. b. TSH, TPOAb, and TGAb were non-normal distributed and were expressed as medians (interquartile range). A rank-sum test was used to detect data differences between the two groups. p < 0.05 was statistically significant.

**Table A2:** The clinical characteristics of patients with Hashimoto’s thyroiditis and controls used in immunohistochemistry

Variable	HT	Control	Reference Range	*P*-value
Number	6	6	—	—
Age^a^	52.34 ± 2.15	53.67 ± 2.56	—	0.487
Gender(M/F)	1/5	1/5	—	—
TSH(mIU/L)^b^	2.66 (1.89, 3.78)	2.24 (1.63, 3.34)	0.35–4.94	1.584
FT4(pmol/L)	12.96 ± 1.74	14.44 ± 2.33	9.01–19.05	1.469
FT3(pmol/L)	3.88 ± 0.62	4.14 ± 0.92	2.63–5.70	1.908
TPOAb(IU/mL)	124(55.15, 352.98)	0.37(0.26, 0.84)	0–5.61	0.000
TgAb(IU/mL)	99.6(25.46, 358)	1.22(0.88, 1.66)	0–4.11	0.000

HT: Hashimoto’s thyroiditis. a. Age, FT4, and FT3 were normally distributed and were expressed as Mean±SDs. Mean comparison between the two groups was performed with the independent sample T-test. b. TSH, TPOAb, and TGAb were non-normal distributed and were expressed as medians (interquartile range). A rank-sum test was used to detect data differences between the two groups. *p* < 0.05 was statistically significant.
